# Case Report: Treatment of Extremely Preterm Infants With Birthweight Below 300 g: Case Series

**DOI:** 10.3389/fped.2021.758683

**Published:** 2021-12-06

**Authors:** Yoshihiko Shitara, Satsuki Kakiuchi, Takeo Mukai, Kohei Kashima, Motohiro Kato, Naoto Takahashi

**Affiliations:** Department of Pediatrics, The University of Tokyo Hospital, Tokyo, Japan

**Keywords:** extremely preterm infants, extremely low birthweight infants, birthweight below 300 g, fetal growth restriction, *en caul* delivery, obstetric complications, parental counseling

## Abstract

Reports on the birth of infants weighing <300 g are quite rare and little is known about the best practices in treating such micropreemies. Therefore, we report here on three cases of low birthweight infants weighing <300 g, of whom two infants survived. The birthweights and gestational ages were ranging 279–293 g and 22 + 6/7 – 23 + 6/7 weeks, respectively. All the infants had severe fetal growth restriction and prematurity. The infant in case 1 died of hepatic rupture, perhaps due to birth trauma, which emphasized the need for less invasive obstetric procedures including *en caul* delivery. The infant in case 2 managed to survive through severe prematurity secondary to hydrops fetalis. However, complications followed soon as tracheal granulation tissue was formed with neurodevelopmental impairment. The infant in case 3 was born recently and her clinical course was less remarkable without severe complications, despite having the least gestational age and birthweight among the three patients. The improved care protocols for extremely low birthweight infants over these years through experiential learning including that with cases 1 and 2 may have ensured the better outcome of case 3. Accumulating evidence and recording the experience of such cases with continuous constructive discussion can contribute to better outcomes and appropriate parental counseling for extremely small babies in the future.

## Introduction

The survival rate of extremely low birthweight infants (ELBWIs) has been improving over the past few decades (1, 2). However, since case reports of infants born with weight <300 g are rare (3–7), we know little about the clinical tips with respect to their treatment and there is a lack of information to provide appropriate parental counseling. In this case study, we share our experiences with three extremely preterm infants with birthweight <300 g including two survivors.

## Case Report

We conducted medical record review at the University of Tokyo Hospital between January 2011 and December 2020. During this period, it was well-accepted in Japan to provide neonatal resuscitation for babies born with gestational age (GA) ≥22 weeks. However, there have been no definitive consensus criteria for resuscitation with respect to birthweight. During this period, there were 10,219 babies ≥22 gestational weeks were born including 82 stillbirths in our hospital. All the stillbirths were intrauterine fetal deaths; thus, no deaths occurred in the delivery room. Three infants weighed <300 g at birth (2.3% of ELBWIs) and their parents hoped for their survival. Neonatal and maternal characteristics are shown in Tables 1, 2, respectively. Neonatal morbidities are given in Table 3. Respiratory management for the patients is shown in Table 4. Information with respect to the clinical courses of the survivors is shown in Table 5.

**Table 1:** Neonatal characteristics.

	**Case 1**	**Case 2**	**Case 3**
Birthweight	293 g (−3.71 SD)	293 g (−2.85 SD)	279 g (−3.29 SD)
Birth length	24.0 cm (−2.85 SD)	22 cm (−3.47 SD)	24.5 cm (−2.14 SD)
Birth head circumference	19.0 cm (−1.74 SD)	19.5 cm (−2.27 SD)	18.0 cm (−1.18 SD)
Gestational age	23 6/7 weeks	23 4/7 weeks	22 6/7 weeks
Outcome	Death	Living	Living
Gender	Male	Male	Female
Apgar score(1 min/5 min)	2/3	1/6	3/5
UApH	NA	6.86	7.18
Size of NG tube	4 Fr	3 Fr	3 Fr
Depth of NG tube	9.0 cm	9.0 cm	9.0 cm
UA catheter	–	–	+
UV catheter	+	+	–
Peripheral arterial catheter	+	+	–
SNAP-II	84	52	24
SNAPPE-II	131	99	71

*NG, nasogastric; UA, umbilical artery; UV, umbilical vein; SNAP, Score for Neonatal Acute Physiology; SNAPPE, SNAP-Perinatal Extension*.

**Table 2:** Maternal characteristics.

	**Case 1**	**Case 2**	**Case 3**
Maternal age	43 y	31 y	40 y
History of pregnancy	G3P0	G1P0	G2P0
Method of conception	ICSI	NC	NC
Singleton birth	+	+	+
Cesarean delivery	+	+	+
Alcohol drinking	–	–	–
Smoking during pregnancy	–	–	–
Smoking before pregnancy	–	–	–
HDP	+	–	+
Hypertension	–	–	+
Oligohydramnios	+	+	–
Diabetes mellitus	–	–	–
PROM	–	–	–
Chorioamnionitis	–	–	–
Antenatal corticosteroids	+	+	+
Antibiotics	+	+	+
Magnesium sulfate	–	–	+
Fetal heart rate monitoring	+	+	+

*G, gravida; P, para; NC, natural conception; ICSI, intracytoplasmic sperm injection; HDP, hypertensive disorders of pregnancy; PROM, premature rupture of membranes*.

**Table 3:** Neonatal morbidities.

	**Case 1**	**Case 2**	**Case 3**
**Major morbidities**
NEC	–	–	–
IVH	–	–	–
Hypotension/Shock	+	+	–
Meconium obstruction of prematurity	–	–	–
Indomethacin for PDA, times	0	1	0
CHD	–	–	–
Pulmonary hemorrhage	–	-	–
Pneumothorax	–	–	–
ROP requiring surgery	–	–	–
Sepsis	–	–	–
Coagulopathy	+	+	–
Liver dysfunction	+	+	–
IFALD	–	+	–
Surgery during hospitalization	–	–	–

*NEC, necrotizing enterocolitis; IVH, intraventricular hemorrhage; PDA, patent ductus arteriosus; CHD, congenital heart disease; ROP, retinopathy of prematurity; IFALD, intestinal failure-associated liver disease*.

**Table 4:** Respiratory management for the patients.

	**Case 1**	**Case 2**	**Case 3**
Endotracheal tube size	2.0 mm	2.0 mm	2.0 mm
Depth of the endotracheal tube	5.0 cm	4.5 cm	5.0 cm
Day of size up to 2.5 mm	–	Day 42	Day 38
Ventilator mode at admission	SIMV	SIMV	SIMV
Day to convert to HFOV	–	Day 0	Day 2
Ventilator mode at extubation	–	HFOV	HFOV
NO	–	+	–
Surfactant, times	0	2	6
Inhaled steroid	–	–	+
Steroid for CLD	–	–	+
Caffeine	–	+	+
CLD 36	–	+	+
Duration of mechanical ventilation	Day 0–3	Day 0–71, 136–164	Day 0–73
Duration of N-CPAP	–	Day 71–82, 128–136	Day 73–89
Duration of HFNC	–	Day 82–128	Day 89–110

*SIMV, synchronized intermittent mandatory ventilation; HFOV, high-frequency oscillatory ventilation; NO, nitric oxide; CLD, chronic lung disease; N-CPAP, nasal-continuous positive airway pressure; HFNC, high-flow nasal cannula*.

**Table 5:** Clinical courses of the survivors.

	**Case 2**	**Case 3**
**Clinical course before discharge**		
Swabbing of colostrum	+	+
Day of starting probiotics	Day 4	Day 2
Day of starting glycerin enema	Day 4	Day 2
Day of starting enteral feeding	Day 5	Day 3
Day of starting human milk supplement	Day 33	Day 29
Mode of feeding at start	Breastmilk	Breastmilk
TPN	Until day 26	Until day 31
Start day of oral feeding	Day 97	Day 101
Blood transfusion	RCC, FFP, PC	RCC, FFP
Dopamine	+	+
Dobutamine	+	+
Vasodilator	–	–
Ultrasound-guided circulatory management	+	+
Hydrocortisone for LCC	–	+
Antibiotics	+	+
HOT	+	+
NG-tube at discharge	–	–
Mode of feeding at discharge	Formula milk	Formula milk
Day of discharge	199 (52 + 0/7 week)	146 (43 + 5/7 week)
Discharge weight	3,126 g	2,570 g
Discharge length	47.2 cm	45.0 cm
Discharge head circumference	36.0 cm	34.2 cm
**Clinical course after discharge**		
Present age	3 y 0 m	0 y 8 m
Weight at 3 years	8.5 kg	
Height at 3 years	82.4 cm	
Head circumference at 3 years	46.0 cm	
KSPD at 3 years		
DQ	28	

*TPN, total parenteral nutrition; RCC, red cell concentrate; FFP, fresh frozen plasma; PC, platelet concentrate; LCC, late circulatory collapse; HOT, home oxygen therapy; NG, nasogastric; KSPD, Kyoto Scale of Psychological Development; DQ, developmental quotient*.

There are no standardized care protocols for extremely premature infants with birthweight below 300 g in Japan. Our standard practice for extremely preterm infants born below 25 weeks of GA is as follows. Endotracheal intubation is performed immediately after birth with a 2.0- or 2.5-mm endotracheal tube. Bovine lung-derived surfactant (*SURFACTEN*^®^ 120 mg/kg/dose) is administrated for respiratory distress syndrome. The initial mode of mechanical ventilation is synchronized intermittent mandatory ventilation (SIMV). When hypoxemia or hypoventilation develops, the ventilation mode is switched to high-frequency oscillatory ventilation (HFOV). Umbilical vessels are the first choice for the arterial line and blood access, considering the skin fragility. A peripherally inserted central catheter (PICC) and/or a peripheral arterial catheter is inserted, if umbilical catheterization is unsuccessful. Dopamine and dobutamine are administrated for systemic hypotension. The first dose of prophylactic indomethacin (0.1 mg/kg, 6 h div) is administered for prevention of intraventricular hemorrhage (IVH) within 6 h after birth, if there are no contraindications such as persistent pulmonary hypertension of the newborn (PPHN). Routine ultrasound is performed every 8 h until 3 days of life (DOL). The second or third dose of the prophylactic indomethacin is withheld, if closure of the arterial duct is confirmed. Parenteral nutrition is started on 0 DOL. The dose of amino acids is initially about 1 g/kg/day, which is gradually increased to 2–3 g/kg/day. Intravenous administration of fatty acids is usually started on 2 to 4 DOL, which is often abandoned due to hyperglycemia. Probiotics and glycerin enema are started on 1–3 DOL. We insist on giving breastmilk as the initial enteral feeding. Routine sedation is not applied; when sedative is needed, usually phenobarbital and, occasionally, fentanyl are given. Blood samples were collected by heel-prick methods after removal of the arterial catheter.

This case study was approved by the Institutional Review Board of our hospital: Ethics Committee of the University of Tokyo Hospital (approval ID 2701).

### Case 1

**Figure 1:**
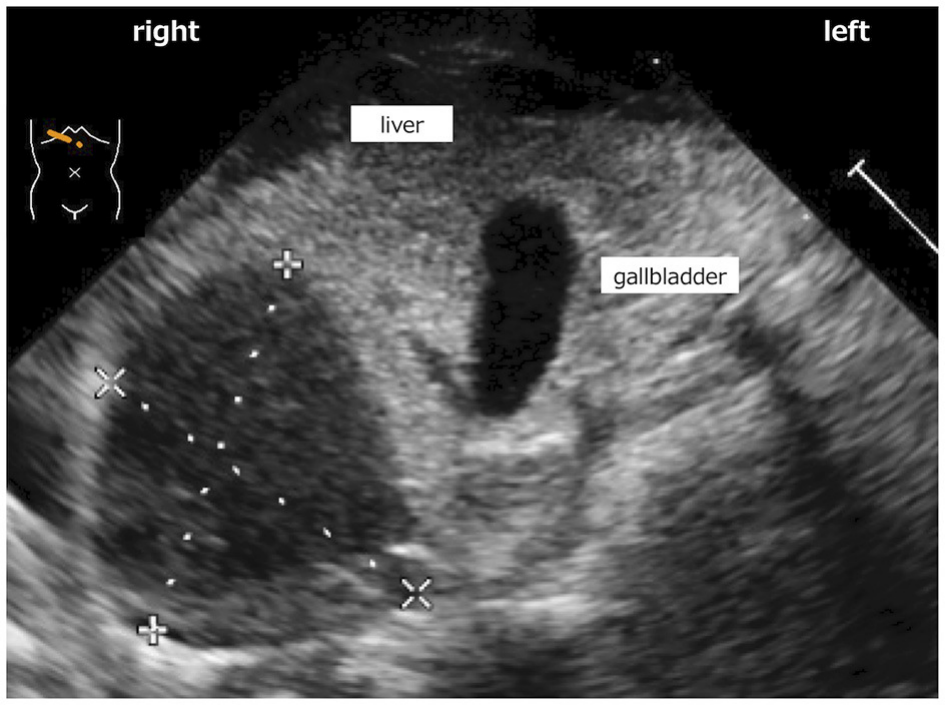
Low echoic lesion in which size was 18 mm × 17 mm that was detected in inferior border of liver on ultrasonography.

A 43-year-old primipara presented with severe hypertensive disorder of pregnancy and fetal growth restriction (FGR) at 19 gestational weeks. At 23 6/7 gestational weeks, an emergency cesarean section was conducted due to worsened hypertensive disorders of pregnancy (HDP) and a non-reassuring fetal status. “En caul” delivery could not be achieved because of the thick uterine wall. The caul refers to the amniotic membrane. To be born in a caul (en caul) means to be born with the head covered by the amnion (or be born within an intact unruptured amnion). A male infant with a birthweight of 293 g was born without any apparent trauma. Endotracheal intubation and surfactant replacement were performed immediately after birth in the delivery room. The umbilical venous catheter (UVC) and the peripheral arterial catheter were successfully placed, while umbilical arterial catheter (UAC) insertion was unsuccessful. Initial examination revealed anemia (hemoglobin level: 10.8 g/dl), disseminated intravascular coagulation (DIC), and the presence of slight ascites on ultrasonography, suggesting intra-abdominal bleeding. Intensive treatment, including HFOV, inotropes, steroids, and massive blood and plasma transfusion for the progressive anemia and DIC, was started. However, liver and adrenal bleeding gradually became evident on ultrasonography (Figure 1) and he continued to suffer from refractory hypotension and further progressive anemia. Throughout the course, his parents hoped to switch to palliative care, seeing his irreversible worsening clinical condition. He died of hemorrhagic shock at 3 DOL.

### Case 2

**Figure 2:**
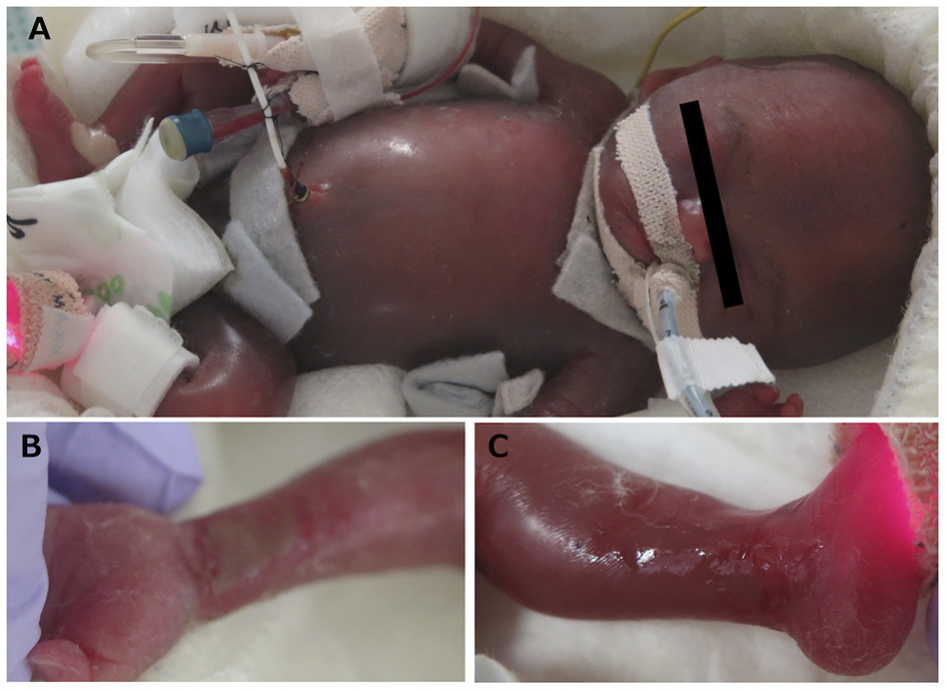
**(A)** Severe general edema was detected by hydrops fetalis. **(B,C)** Desquamation and skin erosions were detected by immaturity.

A 31-year-old primipara was referred to our hospital at 14 gestational weeks due to progressive systemic scleroderma. FGR was noted at 19 gestational weeks and at 23 weeks, progressive hydrops fetalis and non-reassuring fetal status were confirmed. The parents strongly hoped for the survival of the baby; hence, an emergency cesarean section was conducted. A male infant was born at 23 + 4/7 gestational weeks with a birthweight of 293 g via *en caul* delivery. Endotracheal intubation and surfactant replacement were performed immediately after birth in the delivery room. We successfully placed the UVC and the peripheral arterial catheter, whereas the UAC could not be inserted. Severe general edema was detected at birth and continued for a long time (Figure 2). Prophylactic indomethacin was not administered because of severe pulmonary hypertension and inhaled nitric oxide (iNO) was initiated 2 h post-delivery. Patent ductus arteriosus (PDA) became symptomatic at 2 DOL; thus, one dose of indomethacin (0.1 mg/kg) was administered, resulting in PDA closure; iNO was discontinued at 11 DOL. It took almost 2 weeks to stabilize his respiratory and circulatory conditions with HFOV, inotropes, steroids, and repeated blood and plasma transfusions. Mean fluid intake during the first weeks of life was 150.0 ml/kg/day. Breastfeeding started later than 5 DOL because of the severe condition. However, he did not develop necrotizing enterocolitis (NEC). The prophylactic antibiotics and antifungal agents were continued for a month because the skin was extremely fragile with multiple erosions (Figure 2). The skin condition gradually improved with the improvement in nutritional status. The patient was successfully extubated at 71 DOL. However, he was reintubated at 136 DOL due to respiratory distress caused by ventilator-associated subglottic stenosis and required mechanical ventilation until 168 DOL. He was discharged at 199 DOL with home oxygen therapy, without tracheostomy nor tube feeding. He did not develop IVH, periventricular leukomalacia (PVL), or retinopathy of prematurity (ROP) requiring therapy. His ROP was diagnosed as bilateral stage II by the ophthalmologists, but spontaneously resolved. After discharge, the patient underwent endoscopic laser microsurgery for tracheal granulation tissue twice, resulting in total resolution. Home oxygen therapy was discontinued at 1 year of age. The patient is now 3 years old and neurodevelopmental impairment has been identified. He can see, hear, sit, and pull up to standing. He goes wherever he wants by crawling and understands the words of the mother to some extent. He still does not acquire gait or speech, so he is undergoing rehabilitation.

### Case 3

A 40-year-old primipara woman was diagnosed with FGR at 19 gestational weeks. She was transferred to our hospital at 22 gestational weeks due to severe HDP. A female infant was born at 22+6/7 gestational weeks with a birthweight of 279 g after an emergency *en caul* cesarean section due to maternal HDP. Endotracheal intubation and surfactant administration were performed in the delivery room. The UAC and the PICC were successfully placed, while UVC insertion was unsuccessful. Prophylactic indomethacin was administered once, causing PDA closure at 1 DOL. The circulatory status of the patient was successfully stabilized within 72 h of life without IVH. Mean fluid intake during the first weeks of life was 143.0 ml/kg/day. Enteral feeding with breast milk was started at 3 DOL. The breast milk secretion was insufficient; therefore, we increased enteral feeding slowly and started to use hydrolyzed formula milk at 23 DOL. She did not develop NEC throughout her clinical course. Although HFOV, five-time repeated surfactant replacement, and systemic hydrocortisone were required to manage the severe bronchopulmonary dysplasia, she was successfully extubated at 73 DOL. Oral feeding was started at 101 DOL and the patient was discharged at 146 DOL with home oxygen therapy, without tube feeding. She did not develop IVH or PVL. Although ophthalmologists diagnosed her ROP (right; stage II, left; stage III), no therapy was required. She is now 8 months of corrected age and stays healthy in the outpatient clinic without signs of developmental delay; she already sits, rolls over, and pulls up to standing.

## Discussion

We share our experiences with three extremely preterm infants with birthweight <300 g including two survivors. The survival rate of ELBWIs has improved over the past few decades (1, 2). However, the mortality and morbidity rates of babies born with birthweight <500 g remain high. Some studies have reported infants with birthweight <400/500 g in large cohorts; however, the number of infants with birthweight <300 g was very low and the mortality rate was very high (8–10). Per the registrations on the website of University of Iowa currently, there are 41 infants with birthweight <300 g including 13 infants from Japan (11). Detailed clinical information is unavailable, as there have been only a few case reports of infants with birthweight <300 g (3–7). Muraskas et al. reported a female infant weighing 280 g at birth (6). Subsequently, they reported her neurodevelopment at 14 years and long-term follow up of two infants (5, 7). In addition, two cases were reported from Japan (3, 4). Hokuto et al. reported a female infant weighting 290 g at birth in whom they reported the necessity of more than 500 mg/kg of calcium and phosphorus daily intake (4). Arimitsu et al. reported a male infant weighting 268 g at birth, as the tiniest male infant in the world to survive without any serious complications other than severe ROP (3). But, there are no standardized care protocols for extremely premature infants with birthweight below 300 g in Japan and little is known about such small babies to provide appropriate parental counseling. In this case study, we describe three micropreemies.

Case 1 illustrates the fragility of the smallest babies with hepatic rupture. Although we could not determine the exact cause of liver hemorrhage in this case study, fetal birth traumas can occur anytime during and after delivery and this may be one of the limitations associated with their survival. Recently, in the delivery of the preemies, more Japanese obstetricians adopt the “en caul” cesarean section technique, in which the babies are delivered wrapped in amniotic fluids and membranes to avoid birth traumas (12). Additionally, Chia et al. reported that “en caul” cesarean delivery is effective for extremely preterm fetuses to protect them from pressure trauma (13). Such obstetric tips, along with minimal handling in the neonatal intensive care unit (NICU) (14), can alleviate the stress for fragile ELBWIs and may contribute to the improvement of the outcome, although further evaluation is required.

Case 2 was complicated, with the patient having progressive hydrops antenatally and suffering from severe respiratory and circulatory instability after birth. Later, he had tracheal granulation tissue, perhaps due to prolonged intubation and the need for repeated surgery. Although no evidence of PVL or IVH was found, neurodevelopmental impairment was evident during follow-up. Hence, it was surmised that achieving intact survival in the most severe micropreemies still remains critically challenging.

Case 3 was born most recently. Her clinical course was less remarkable without severe complications despite having the lowest GA and the smallest birthweight among the three patients. The accumulation of experience on ELBWIs over these years, including that with cases 1 and 2, may have played a role in improving the outcome. Her neurological prognosis is yet unknown and we will continue to follow-up.

Each case had difficulties with respect to blood access due to the small size of the infants. In cases 1 and 2, UAC insertion was unsuccessful and in case 3, the UVC was unavailable. In case 1, an arterial catheter could be placed, but the wave of the artery was not displayed because of imperfect circulation. For case 2, the peripheral arterial line was placed with difficulties, but the procedure worsened the skin erosions. For case 3, the peripherally inserted central venous catheters, not the UVC, were successfully placed and the clinical course was uncomplicated. More secure and less invasive blood access procedures are required for the smallest preemies. However, the small medical devices available are limited. For example, from 2.5 to 5.0 Fr single lumen UAC are available in our hospital. On the other hand, available double-lumen UVC is only one size. For endotracheal tube, the inner diameter of the smallest one available is 2.0 mm (15, 16). However, we somehow managed to intubate these three cases with the smallest tube. Nonetheless, the thinner the tube, the higher the airway resistance gets, which causes unignorable disadvantages in the respiratory management (17).

There are many arguments about the ethical aspect, considering the long-term outcome. Currently, there are many options for the treatment in the NICU: surfactant, HFOV, iNO, cyclooxygenase inhibitors for PDA, minimally invasive PDA surgery, ultrasound-guided circulatory management, prophylaxis of IVH with indomethacin, probiotics, etc. (18–21). All of these have contributed to the short-term survival of ELBWIs. On the other hand, many unsolved problems remain: NEC, cholestasis, neurodevelopmental impairment, short stature, risk for lifestyle-related diseases, etc. (7, 22–26). Not only the lung and brain, but also the gastrointestinal tract, liver, and kidney should be restored for the following life. The answers to the question “How small is too small?” should always change with time and place, according to the medical standard, socioeconomic status, culture of each region, and so on (27–32). In Japan, an approach of universal resuscitation is favored for neonates at 22 and 23 weeks of gestation, although the definitive criteria on the threshold of birthweight for resuscitation remain unestablished. In such a situation, Seri et al. reported about the limit of viability as birthweight falls <500 g or the pregnancy is at <23 weeks of gestation (27). Similarly, the 2010 American Heart Association guidelines on neonatal resuscitation proposed that the threshold for withholding resuscitation might be GA <23 weeks or birthweight <400 g (29–31). Leah et al. reported that neither selective nor universal resuscitation at 22 weeks of gestation is cost-effective compared with no resuscitation (32).

Further accumulation of experience on these cases and continuation of constructive discussion are required.

## Data Availability Statement

The raw data supporting the conclusions of this article will be made available by the authors, without undue reservation.

## Ethics Statement

This case study was approved by the Ethics Committee of the University of Tokyo Hospital (Approval ID 2701). The parents of patients provided their written informed consent for participation in this case study and a written informed consent was obtained from the parents of the infants for the publication of this case study with any identifiable data or images.

## Author Contributions

YS was in charge of these case studies and wrote the first draft of manuscript. SK and TM were in charge of the patients in this case study and also supervised the drafting, reviewed, and revised the manuscript. KK and MK took care of the patients and provided access to the patients and also helped to write the draft. NT supervised the data analysis and critically reviewed the manuscript. All the authors approved the final manuscript for submission.

## Conflict of Interest

The authors declare that the research was conducted in the absence of any commercial or financial relationships that could be construed as a potential conflict of interest.

## Publisher's Note

All claims expressed in this article are solely those of the authors and do not necessarily represent those of their affiliated organizations, or those of the publisher, the editors and the reviewers. Any product that may be evaluated in this article, or claim that may be made by its manufacturer, is not guaranteed or endorsed by the publisher.
